# Supplementary material for: Improving Interaction at Polymer–Filler Interface: The Efficacy of Wrinkle Texture
Source: Nanomaterials (Basel). 2020 Jan 25;10(2):208. doi: 10.3390/nano10020208 (PMC7074972; doi:10.3390/nano10020208)
Supplement: Supplementary file 1 [file nanomaterials-10-00208-s001.pdf]

nanomaterials

Article

Improving interaction at polymer-filler interface: the efficacy of wrinkle texture

Pietro Russo[1], Virginia Venezia[2], Fabiana Tescione[3], Joshua Avossa[4], Giuseppina Luciani[2], Brigida Silvestri[2]*, Aniello Costantini[2]

[1]Institute for Polymers, Composites and Biomaterials, National Research Council, via Campi Flegrei 34, 80078 Pozzuoli-Naples, Italy

[2] Department of Chemical, Materials and Production Engineering, University of Naples "Federico II", p.le V. Tecchio 80, 80125 Naples, Italy

[3]Institute for Polymers, Composites and Biomaterials, National Research Council, Portici, Naples, Italy

[4] Institute of Atmospheric Pollution Research-National Council (IIA-CNR), Research Area of Rome 1, via Salaria Km 29.300, Monterotondo, 00016, Italy

*Corresponding author email: brigida.silvestri@unina.it, tel.: +390817682413; fax: +390817682595.

*Corresponding author: tel.: +390817682413; fax: +390817682595. E-mail address: brigida.silvestri@unina.it

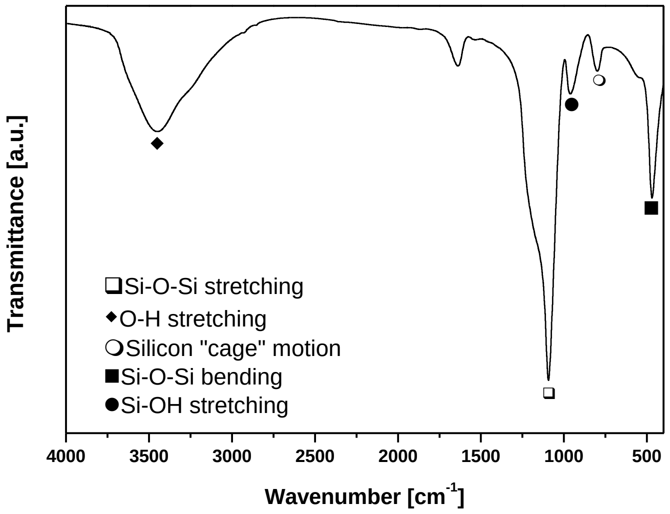

Figure S1. *FT-IR spectrum of SiO_2_w NPs.*

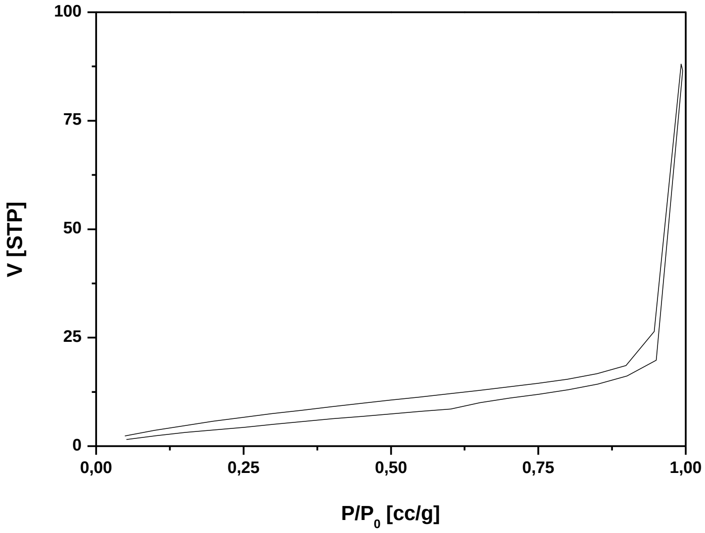

Figure S2. Adsorption isotherms at 77K of Stöber NPs.

*Corresponding author: tel.: +390817682413; fax: +390817682595. E-mail address: brigida.silvestri@unina.it

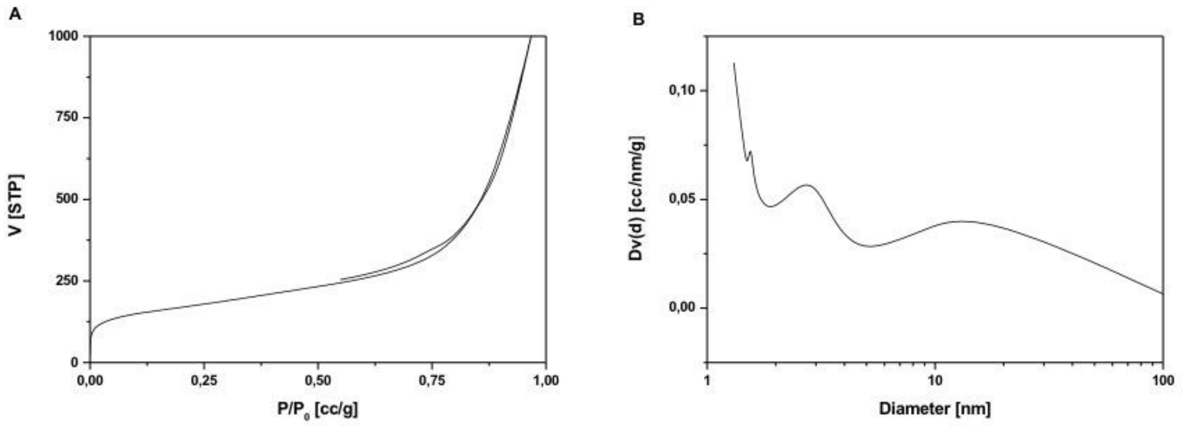

Figure S3. Adsorption isotherms at 77K (A) and pore size distribution (B) of wrinkled NPs.

*Corresponding author: tel.: +390817682413; fax: +390817682595. E-mail address: brigida.silvestri@unina.it
